# Rattle Snake Bite

**Published:** 1885-03

**Authors:** 


### RATTLE SNAKE BITE.

"Perhaps there is no place on this continent where rattle snakes congregate in larger numbers than along the Tioga River, in Pennsylvania. The mountains through which that crooked stream finds a passage are the homes of the rattle snake, and in the warm summer months they leave their dens by thousands for the narrow valley, where they bask in the sun for a few weeks, and those that do not get their heads bruised by the heel of man, or get cut in pieces by lying nights on the warm rails of the Corning and Blossburg Railroad, as thousands of them are, crawl back to their rocky ledges. In midsummer it is an almost daily occurence for some one to be bitten by these reptiles in the Tioga Valley, and domestic animals are frequently bitten. But the residents of this region do not look upon the bite of these reptiles as anything serious. When a person is bitten, a handful of mud or wet clay is applied to the wound, and fresh applications are made every ten or fifteen minutes until the poison is all drawn out. The first thing a native of that locality does after being bitten is to seek a place where hogs wallow, where soft mud or wet clay is always found. He binds on a quantity and is soon cured.

The same is applied to domestic animals when they exhibit symptoms of snake bite, and this treatment is always successful if the application is made before the poison has gone too far. It is well known that hogs are not harmed by snake bites, and this is accounted for by the porkers' habit of cooling off by lying in a mud hole.

A farmer on the line of the Corning and Blossburg Railroad had a small cur dog that made it his business to kill rattle snakes. In his encounters with large ones he would be bitten several times, but as soon as the fight was over he would hasten to the farm yard and lay himself in a "hog wallow" for several hours, when he would come out entirely free from poison.

It is well to fortify the system with stimulants, then apply the clay mud, and cure is certain. The clay must be moist, and or of the consistence of dough."

We are inclined to think that the snakes referred to were of a different breed, or age, or condition from some of those whose bite is fatal. On the 13th inst., in this city, James Reilly, a snake charmer, who was exhibiting rattle snakes, and had taken one of them out of its box, according to custom, was bitten in the right hand by the reptile. The man returned the snake to the box and began at once to suck the poison from the wound with his mouth, then swallowed a quart of whisky and went to the hospital. Here he was treated with hypodermic injections of whisky and sedatives; other remedies were also tried, but it was of no avail. After lingering for over twenty-fours in agony, the unfortunate man died.

Reilly himself declared his belief that the bite of that particular kind of rattle snake—diamond back—was always fatal.

A few hours before Reilly died a Westchester County farmer named Purdy appeared, bringing what he claimed to be a sure cure, as it had saved the lives of at least fifty persons. He was allowed to try the remedy upon the sick man. It consisted of a decoction of herbs. The only effect was to procure a short sleep.—*Scientific American.*

